# A single dose, BCG-adjuvanted COVID-19 vaccine provides sterilising immunity against SARS-CoV-2 infection

**DOI:** 10.1038/s41541-021-00406-4

**Published:** 2021-11-30

**Authors:** Claudio Counoupas, Matt D. Johansen, Alberto O. Stella, Duc H. Nguyen, Angela L. Ferguson, Anupriya Aggarwal, Nayan D. Bhattacharyya, Alice Grey, Owen Hutchings, Karishma Patel, Rezwan Siddiquee, Erica L. Stewart, Carl G. Feng, Nicole G. Hansbro, Umaimainthan Palendira, Megan C. Steain, Bernadette M. Saunders, Jason K. K. Low, Joel P. Mackay, Anthony D. Kelleher, Warwick J. Britton, Stuart G. Turville, Philip M. Hansbro, James A. Triccas

**Affiliations:** 1 School of Medical Sciences, Faculty of Medicine and Health, The University of Sydney, Camperdown, NSW Australia; 2 Tuberculosis Research Program at the Centenary Institute, The University of Sydney, Sydney, NSW Australia; 3 Centre for Inflammation, Centenary Institute and University of Technology Sydney, Faculty of Science, School of Life Sciences, Sydney, NSW Australia; 4 Kirby Institute, University of New South Wales, Sydney, NSW Australia; 5 Department of Clinical Immunology, Royal Prince Alfred Hospital, Sydney, NSW Australia; 6 RPA Virtual Hospital, Sydney Local Health District, Sydney, NSW Australia; 7 School of Life and Environmental Sciences, The University of Sydney, Sydney, NSW 2006 Australia; 8 Sydney Institute for Infectious Diseases and Charles Perkins Centre, The University of Sydney, Camperdown, NSW Australia

**Keywords:** Protein vaccines, Experimental models of disease

## Abstract

Global control of COVID-19 requires broadly accessible vaccines that are effective against SARS-CoV-2 variants. In this report, we exploit the immunostimulatory properties of bacille Calmette-Guérin (BCG), the existing tuberculosis vaccine, to deliver a vaccination regimen with potent SARS-CoV-2-specific protective immunity. Combination of BCG with a stabilised, trimeric form of SARS-CoV-2 spike antigen promoted rapid development of virus-specific IgG antibodies in the blood of vaccinated mice, that was further augmented by the addition of alum. This vaccine formulation, BCG:CoVac, induced high-titre SARS-CoV-2 neutralising antibodies (NAbs) and Th1-biased cytokine release by vaccine-specific T cells, which correlated with the early emergence of T follicular helper cells in local lymph nodes and heightened levels of antigen-specific plasma B cells after vaccination. Vaccination of K18-hACE2 mice with a single dose of BCG:CoVac almost completely abrogated disease after SARS-CoV-2 challenge, with minimal inflammation and no detectable virus in the lungs of infected animals. Boosting BCG:CoVac-primed mice with a heterologous vaccine further increased SARS-CoV-2-specific antibody responses, which effectively neutralised B.1.1.7 and B.1.351 SARS-CoV-2 variants of concern. These findings demonstrate the potential for BCG-based vaccination to protect against major SARS-CoV-2 variants circulating globally.

## Introduction

The world has entered a critical stage in the continuing fight against COVD-19. The deployment of effective vaccines has had a profound impact in reducing cases and SARS-CoV-2 transmission in countries with high vaccine coverage^1,2^. However global cases are again on the rise, due to the emergence of new SARS-CoV-2 variants that exhibit increased transmissibility^3,4^ and partial immune escape^5,6^. For example, vaccination with one of the most widely deployed vaccines, ChAdOx1 nCoV-19, does not protect against mild-to-moderate COVID-19 due to the B.1.351/beta variant in South Africa^7^. A critical issue is ensuring the adequate supply of vaccines to the hardest-hit low and middle-income countries, particularly due to complex logistical requirements (e.g. storage at low temperature for mRNA vaccines). The requirement for multiple doses for most approved vaccines is a barrier to rapid, mass vaccination and has necessitated changes in dosing schedules in some countries to ensure sufficient vaccine coverage^8^. Thus, ensuring the global supply of vaccines effective against emerging variants will be necessary to control the global COVID-19 pandemic.

One unique strategy is to ‘repurpose’ existing licensed vaccines for use against COVID-19. Significant interest has focussed on *Mycobacterium bovis* bacille Calmette-Guerin (BCG), the tuberculosis (TB) vaccine. Considerable data have been accumulated to show that BCG has beneficial, non-specific effects on immunity that affords protection against other pathogens, particularly respiratory infections^9^. Most recently, BCG vaccination was shown to protect against viral respiratory tract infections in the elderly (>65 years old) with no significant adverse events^10^. This non-specific protective effect is attributed to the ability of BCG to induce ‘trained immunity’ i.e. reprogramming of innate immune responses to provide heterologous protection against disease. For these reasons, a number of randomised controlled trials have commenced to determine if BCG vaccination/re-vaccination can reduce the incidence and severity of COVID-19^9,11^. While these and other trials will determine if BCG can reduce the impact on COVID-19 during the current pandemic, BCG does not express SARS-CoV-2-specific antigens and thus, would not induce long-term immune memory.

Here, we have exploited the immunostimulatory properties of BCG to develop a SARS-CoV-2 vaccine, BCG:CoVac, that combines a stabilised, trimeric form of the spike protein with the alum adjuvant. BCG:CoVac stimulated SARS-CoV-2-specific antibody and T cell responses in mice after a single vaccination, including the elicitation of high-titre NAbs. Critically, a single dose was shown to protect mice against severe SARS-CoV-2, demonstrating that BCG:CoVac is a highly immunogenic and promising vaccine candidate.

## Results

### BCG vaccination promotes SARS-CoV-2-specific antibody and T cell responses in mice

**Fig. 1:**
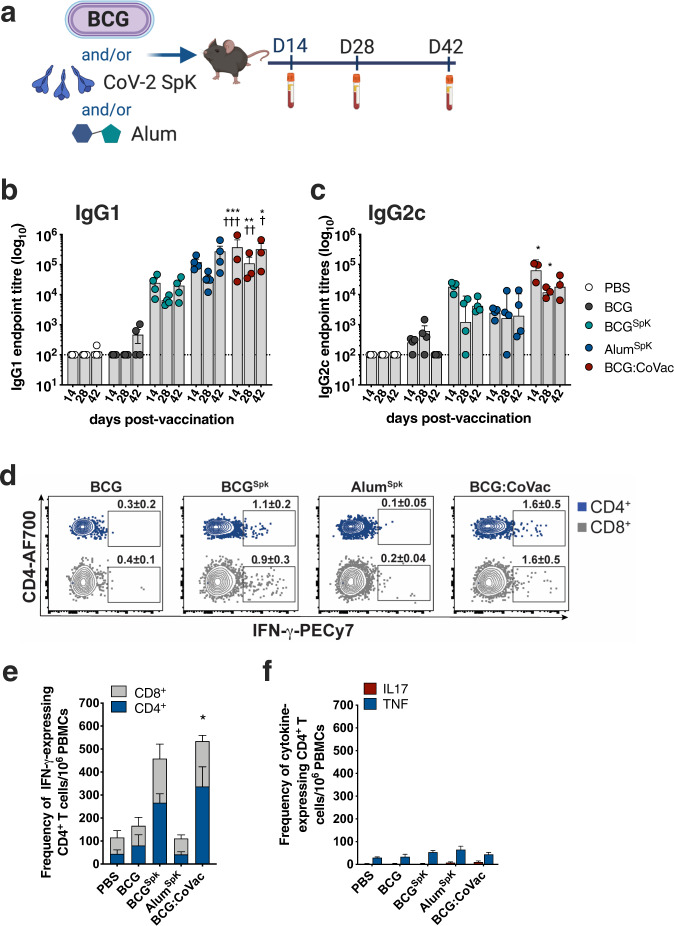
Single immunisation with BCG:CoVac vaccine induces rapid development of anti-SARS-CoV-2 spike antibodies and IFN-γ-secreting T cells. **a** C57BL/6 mice (*n* = 4/group) were vaccinated subcutaneously with PBS, BCG, BCG^SpK^, Alum^SpK^ or BCG:CoVac and whole blood collected at day 14, 28 and 42. **b**, **c** Spike-specific IgG1 and IgG2c titres in plasma were determined by ELISA and estimated by the sigmoidal curve of each sample interpolated with the threshold of the negative sample ±3 standard deviations. The dotted line shows the limit of detection. **d** At 14 days post-vaccination PBMCs were restimulated ex vivo with 5 μg/mL of SARS-CoV-2 spike and cytokine production determined by flow cytometry. Representative dot plots of CD44^+^ CD4^+^ T cells and CD44^+^ CD8^+^ T cells expressing IFN-γ with average ± SEM (gating strategy in Supplementary Fig. 3). **e** Frequency of circulating CD4^+^ and CD8^+^ T cells expressing IFN-γ or **f** CD4^+^ T cells expressing IL-17 or TNF per 10^6^ PBMCs. Data presented as mean ± SEM and is representative of two independent experiments. Significant differences between groups compared to BCG^Spk^ *p < 0.05, **p < 0.01, ***p < 0.01 or Alum^Spk^^†^*p* < 0.05, ^††^*p* < 0.01, ^†††^*p* < 0.001 were determined using one-way ANOVA.

The immunostimulatory properties of BCG^12^ led to us to test if the vaccine could serve as the backbone for a unique vaccine platform against COVID-19. This was also supported by our observation that prior BCG immunisation could augment anti-spike IgG responses after boosting with SpK formulated in Alhydrogel/alum (Alm^SpK^) (Supplementary Fig. 1). To determine if this property of BCG could be used in a single vaccine formulation, we subcutaneously (s.c) vaccinated mice with a single dose of BCG formulated with a stabilised, trimeric form of the SARS-CoV-2 spike protein^13^ and the titre of plasma IgG2c or IgG1 anti-SpK antibodies was determined at various timepoints post-immunisation (Fig. 1a). While BCG vaccination resulted in background levels of anti-SpK antibodies, titres were approximately 100-fold higher for both antibody isotypes after BCG^Spk^ vaccination, and similar to those levels achieved with Alm^SpK^ (Fig. 1b, c). Addition of alum to BCG^Spk^ (termed BCG:CoVac) further increased antibodies titres, particularly IgG2c, which were significantly greater after BCG:CoVac vaccination compared to mice immunised with either BCG or Alm^SpK^, at all timepoints examined (Fig. 1b, c).

The IgG2c Ab isotype correlates with Th1-like immunity in C57BL/6 mice^14^, and such responses are considered necessary for effective protection against SARS-CoV-2 infection^15^. We therefore examined the frequency of IFN-γ-expressing T cells after a single dose of BCG:CoVac at 2 weeks post-vaccination. BCG^SpK^ and BCG:CoVac induced the generation of SpK-specific CD4^+^ and CD8^+^ T cells secreting IFN-γ (Fig. 1d, e), consistent with Th1 immunity observed after BCG vaccination^16^. The greatest response was observed after vaccination with BCG:CoVac, with the numbers of IFN-γ-secreting T cells significantly increased compared to vaccination with either BCG or Alum^SpK^. Low levels of the inflammatory cytokines IL-17 and TNF were observed after BCG:CoVac vaccination (Fig. 1f).

**Fig. 2:**
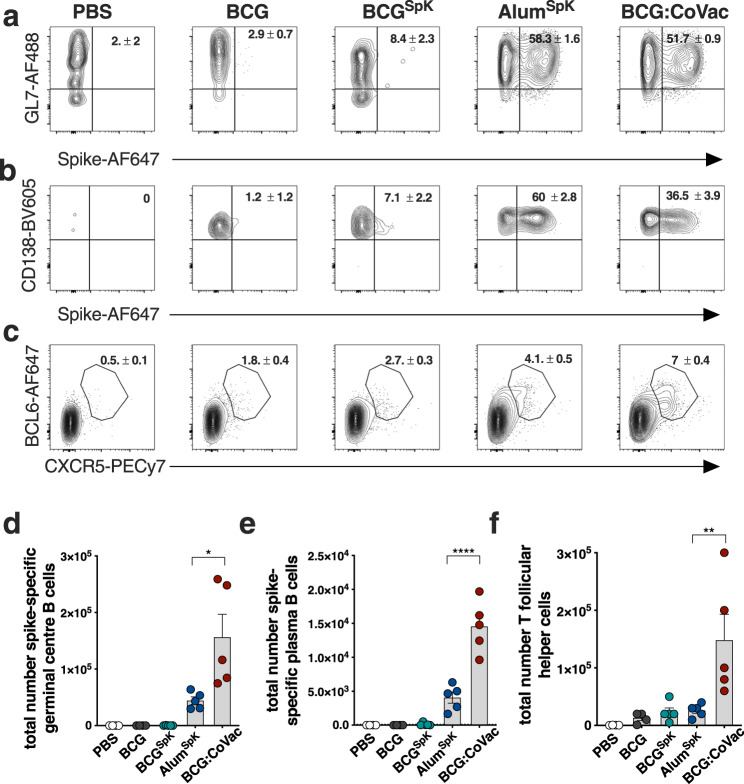
BCG:CoVac vaccination promotes expansion of T follicular helper cells and spike-specific B cells in mice. **a**–**c** C57BL/6 mice (*n* = 5/group) were vaccinated subcutaneously with PBS, BCG, BCG^SpK^, Alum^SpK^ or BCG:CoVac and 7 days after immunisation B and T cell response assessed by multicolour flow cytometry in the draining lymph node. Shown are representative dot plots of spike-specific germinal centre B cells (**a**, CD19^+^MHCII^+^GL7^+^CD38^−^), plasma B cells (**b**, CD19^+^MHCII^+^CD138^+^) and T follicular helper T cells (**c**, CXCR5^+^BCL6^+^) with average ±SEM (gating strategy in Supplementary Figs. 4 and 5). **d**–**f** The total number of **d** spike^+^ GC B cells, **e** spike^+^ plasma cells and **f** T follicular helper cells. Data presented as mean ± SEM and is representative of two independent experiments. Significant differences between groups *p < 0.05, **p < 0.01, ***p < 0.01 were determined by one-way ANOVA.

We further dissected vaccine-induced immunity by defining the cellular composition in draining lymph nodes 7 days after vaccination. Both Alum^SpK^ and BCG:CoVac induced appreciable expansion of SpK-specific germinal centre (GC) B cells (CD19^+^MHCII^+^GL7^+^CD38^-^; Fig. 2a) and plasma B cells (CD19^+^MHCII^+^CD138^+^; Fig. 2b). Cells with a T follicular helper cell (Tfh) phenotype (CD4^+^CXCR5^+^BCL6^+^) were apparent after vaccination with Alum^SpK^ or BCG:CoVac, with Tfh frequency greatest in the latter group (Fig. 2c). The total numbers of GC B cells (Fig. 2d), plasma B cells (Fig. 2e) and Tfh cells (Fig. 2f) were all significantly increased in BCG:CoVac-vaccinated mice compared to immunisation with Alum^SpK^.

Overall, these data show that co-delivery of trimeric SpK antigen with BCG vaccination promotes early and pronounced anti-SARS-CoV-2 immunity, and this is further enhanced with the addition of alum.

### High-titre, SARS-CoV-2 neutralising antibodies after a single immunisation with BCG:CoVac

**Fig. 3:**
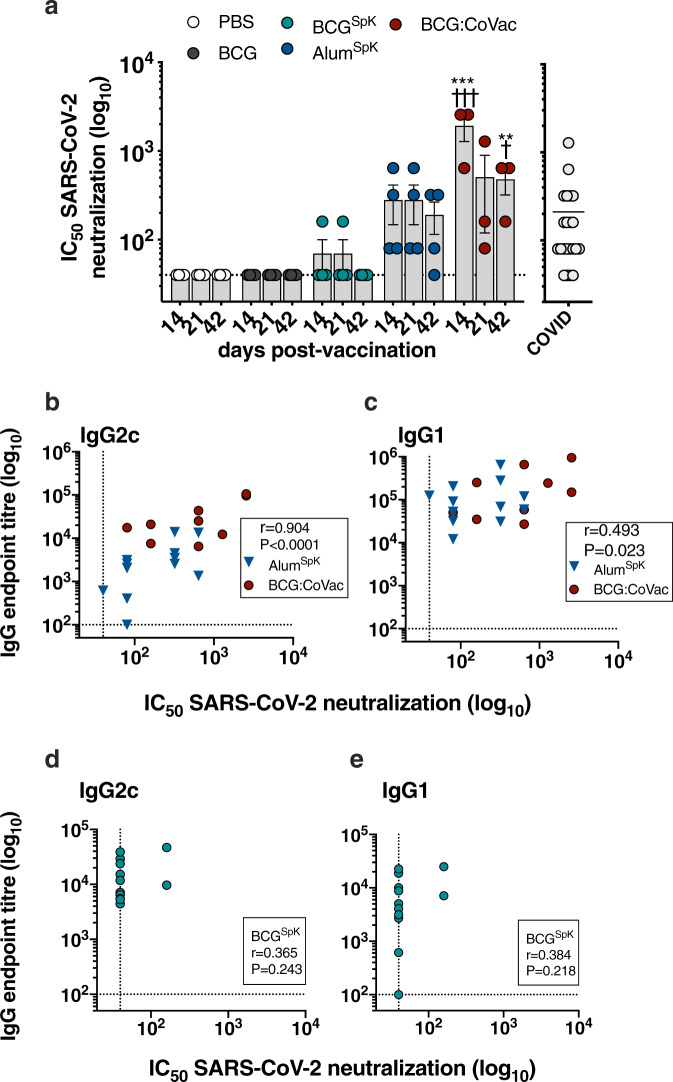
BCG:CoVac induces high-titre neutralising antibodies against live SARS-CoV-2 that correlate with the production of antigen-specific IgG2c. **a**–**e** Plasma from vaccinated mice (from Fig. 1) were tested for neutralising activity against live SARS-CoV-2 infection of VeroE6 cells. **a** Neutralising antibody (NAb) titres (IC_50_) were calculated as the highest dilution of plasma that still retained at least 50% inhibition of infection compared to controls. NAb titres from PCR confirmed SARS-CoV-2-infected individuals (COVID) were determined using the same method. **b**, **c** Spearman correlations of spike-specific IgG2c or IgG1 titres and NAbs after Alum^SpK^ or BCG:CoVac vaccination. **d**, **e** Correlation of IgG2c or IgG1 titres and NAbs after vaccination with BCG^SpK^. The dotted line shows the limit of detection. Data presented as mean ± SEM and is representative of two independent experiments. Significant differences between groups compared to BCG^Spk^ **p < 0.01, ***p < 0.0 or Alum^Spk^^†^*p* < 0.05, ^†††^*p* < 0.001 was determined by one-way ANOVA.

The elicitation of GC B cell and Tfh responses after immunisation with experimental SARS-CoV-2 vaccines correlate strongly with the induction of neutralising antibodies (NAbs)^17^. Such NAbs are a key determinant of protection induced by current vaccines used in humans^18^. We therefore measured NAb levels after a single dose of BCG:CoVac. No NAbs were detected in the plasma of mice vaccinated with BCG (Fig. 3a). Surprisingly, NAb titres were at near background levels for mice vaccinated with BCG^SpK^ (Fig. 3a), despite the high levels of IgG Ab isotypes detected in these same animals (Fig. 1). High NAb titres were detected as early as 2 weeks post-immunisation upon vaccination with BCG:CoVac, and titres were significantly increased compared to vaccination with Alum^SpK^ (~10-fold increase). The mean NAb titres in the plasma of BCG:CoVac-vaccinated mice were ~10-fold greater than those seen in SARS-CoV-2-infected humans (Fig. 3a). Although the levels of NAbs peaked at 2 weeks post-vaccination with BCG:CoVac, they remained significantly elevated up to day 42 post-immunisation unlike those in the other immunised groups.

Since previous work suggests that the level of IgG antibody correlates with NAb titres after SARS-CoV-2 infection^19^, we examined whether a similar phenomenon was observed after vaccination with BCG:CoVac. Strong correlation (*r* > 0.9) was observed between IgG2c isotype and NAbs in groups vaccinated with BCG:CoVac or Alum^SpK^ (Fig. 3b), with a significant yet less robust correlation between IgG1 and NAbs for these groups (Fig. 3c). There was no correlation between NAbs and either IgG1 or IgG2c Ab for mice vaccinated with BCG^SpK^ alone (Fig. 3d, e).

### BCG:CoVac affords sterilising immunity against SARS-CoV-2 infection in K18-hACE2 mice

**Fig. 4:**
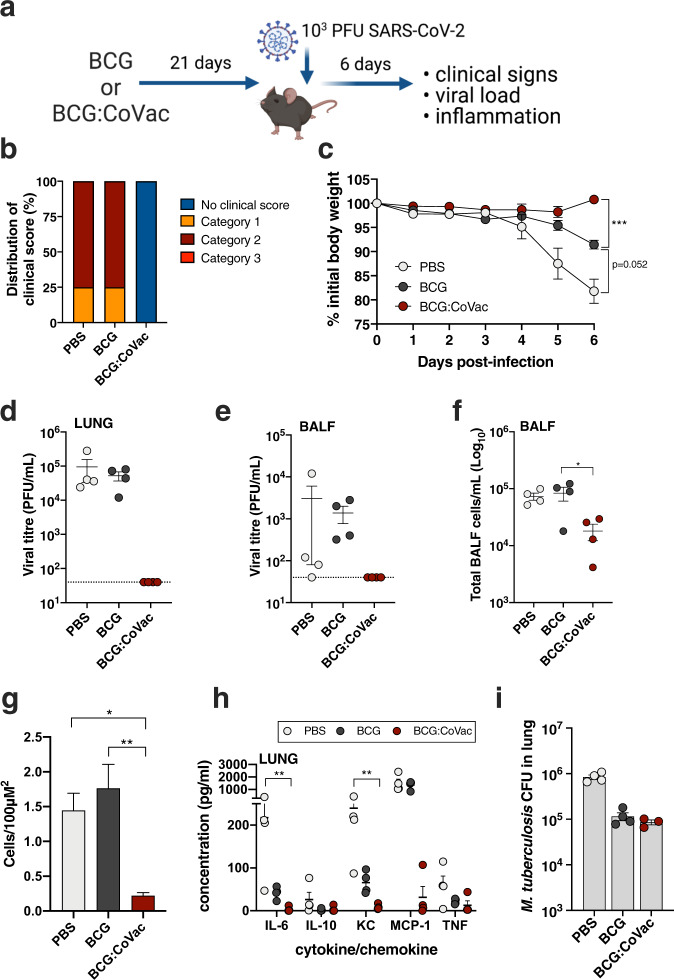
A single dose of BCG:CoVac protects against severe SARS-CoV-2 infection. **a** Male K18-hACE2 mice (n = 4/group) were immunised with sham (PBS), BCG or BCG:CoVac 21 days prior to challenge with 10^3^ PFU SARS-CoV-2. Disease outcomes were assessed 6 days later. **b** Clinical scores at day 6 post-infection. **c** Percentage of initial body weight loss in K18-hACE2 mice. Viral titres in lung homogenates (**d**) or bronchoalveolar lavage fluid (BALF) (**e**) were determined using plaque assay. The dotted line represents the limit of detection. **f** Total inflammatory cells in bronchoalveolar lavage fluid (BALF). **g** Total number of inflammatory cells in stained histological sections of lungs. **h** Cytokine/chemokine quantification in lung homogenates. **i** Six weeks after immunisation mice were challenged with *M. tuberculosis* H37Rv by aerosol (~100 CFU) and four weeks later the bacterial load was assessed in the lungs and presented as log_10_ of the mean CFU ± SEM. Significant differences between groups *p < 0.05, **p < 0.01 were determined by one-way ANOVA.

Wild-type mice are not permissive to SARS-CoV-2 infection, owing to incompatibility in the receptor binding domain of the viral spike protein with the murine angiotensin-converting enzyme 2 (ACE2)^20^. Transgenic mice expressing human (h)ACE2 such as the K18-hACE2 mouse, are highly susceptible to SARS-CoV-2 infection, succumbing to lethal infection within 7 days post-infection^21^. We therefore assessed the protective role of BCG or BCG:CoVac vaccination in SARS-CoV-2 infection in K18-hACE2 mice. Mice were vaccinated 21 days prior to inoculation with 10^3^ PFU SARS-CoV-2 (Fig. 4a). Mice sham vaccinated with PBS succumbed to infection within 6 days with substantial deterioration in their condition with high clinical scores (Fig. 4b) and 20% weight loss (Fig. 4c). This outcome was associated with high viral titres in the lung tissues (Fig. 4d) and airways (bronchoalveolar lavage fluid, BALF) (Fig. 4e). These events led to extensive lung inflammation with substantial increases in inflammatory cells in the airways (Fig. 4f) and lung tissue (Fig. 4g), and the levels of the pro-inflammatory cytokine, IL-6, and chemokines KC (murine equivalent of IL-8) and MCP-1, in the lung tissues (Fig. 4h) and airways (Supplementary Fig. 2). MCP-1 was also increased in serum (Supplementary Fig. 2). These are the archetypal cytokines associated with severe human COVID-19^22^. Vaccination with BCG showed some beneficial effects and partially protected against weight loss (~10%) and lung IL-6 and KC responses but not in other disease features. Remarkably, vaccination with BCG:CoVac 21 days prior to infection completely protected against infection, with no observable weight loss or any clinical scores throughout the duration of the experiment (Fig. 4b, c). These mice had no detectable virus in the airways or lungs (Fig. 4d, e). They had few signs of lung inflammation with moderate levels on inflammatory cells in the airways and virtually none in the lung tissue (Fig. 4g), and only baseline levels of all pro-inflammatory cytokines in the airways, lung and serum (Fig. 4h and Supplementary Fig. 2). Importantly, combination of the spike protein and alum with BCG did not alter the protective efficacy of the BCG vaccine against aerosol *M. tuberculosis* in mice (Fig. 4i).

Collectively, these findings demonstrate that single dose administration of BCG:CoVac is sufficient to completely protect mice from the development of COVID-19 disease manifestations, and to neutralise infectious SARS-CoV-2 and prevent pathogenic inflammation in the lung.

### Enhancing BCG:CoVac immunity against SARS-CoV-2 by heterologous vaccine boosting

**Fig. 5:**
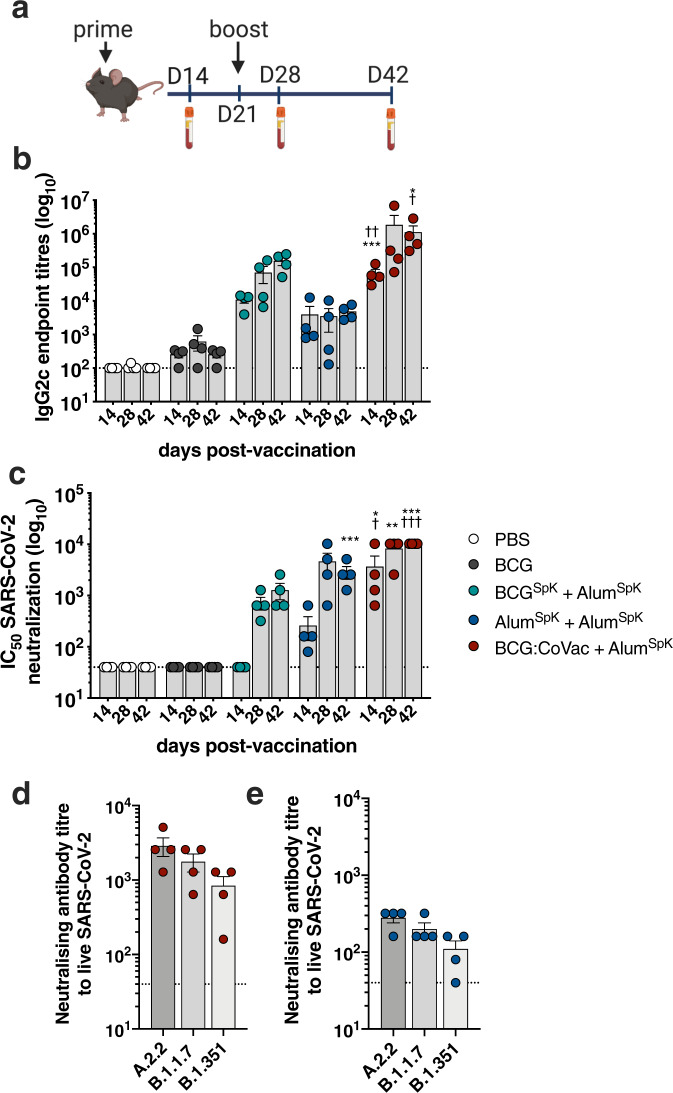
Heterologous boosting of BCG:CoVac-primed mice results in augmented SARS-CoV-2-specific IgG2c titres and neutralising antibodies. **a** C57BL/6 mice (*n* = 3–4/group) were vaccinated (as in Fig. 1) and at day 21 mice were boosted with Alum^Spk^. **b** Spike-specific IgG2c titres in plasma were determined by ELISA estimated from the sigmoidal curve of each sample interpolated with the threshold of the negative sample ±3 standard deviations. **c** Neutralising antibody (NAb) titres (IC_50_) were calculated as the highest dilution of plasma for all groups that still retained at least 50% inhibition of infection compared to controls. The dotted line shows the limit of detection. **d**, **e** NAb titres against the B.1.1.7 or B.1.351 SARS-CoV-2 variants were also determined using plasma from either **d** Alum^SpK^ or **e** BCG:CoVac-vaccinated mice. Data presented as mean ± SEM and is representative of two independent experiments. Significant differences between groups compared to BCG^Spk^ *p < 0.05, **p < 0.01, ***p < 0.01 or Alum^Spk^^†^*p* < 0.05, ^††^*p* < 0.01, ^†††^*p* < 0.001 were determined by one-way ANOVA.

COVID-19 subunit vaccines typically display poor immunity after a single dose and require a booster to induce sufficient generation of NAbs^23^. Whilst we observed high-titre NAbs as early as two weeks post-BCG:CoVac vaccination (Fig. 3), we sought to determine if responses could be further augmented by boosting BCG:CoVac 3 weeks later with a prototype subunit vaccine (Alum^SpK^) (Fig. 5a). At 7 days post-boost (day 28), IgG2c titres in plasma from mice primed either with BCG^SpK^ or BCG:CoVac were increased and remained elevated up to day 42 (Fig. 5b). Corresponding augmentation of NAbs was also seen in these boosted groups, with significantly elevated responses in BCG:CoVac-primed mice boosted with Alum^SpK^ (Fig. 5c). Boosting Alum^SpK^ vaccination with a second dose led to a greater than 10-fold increase in NAbs in boosted mice; however, responses were significantly higher in those with the BCG:CoVac-prime, Alum^SpK-^boost combination (Fig. 5c). Plasma from BCG:CoVac-vaccinated mice was able to neutralise both the B.1.1.7 variant (1.3-fold decrease compared to wild-type virus) and B.1.351 variant (2.7-fold decrease) (Fig. 5d). Neutralisation capacity against B.1.1.7 and B.1.351 was maintained to some extent after prime-boost with Alum^SpK^ only, however titres were approximately 10-fold less than those following the BCG:CoVac prime, Alum^SpK^ combination (Fig. 5e).

Taken together, these data indicate that the antigen-specific immunity imparted by BCG:CoVac can be further enhanced by heterologous boosting with a second SARS-CoV-2 vaccine, with this vaccination regimen able to induce antibodies that can neutralise key VOCs.

## Discussion

Global vaccine access and distribution to low- and middle-income countries is critical for the control of the COVID-19 pandemic. Vaccines must offer effective protective immunity yet should be cheap to manufacture and have feasible cold chain management requirements. This study describes a COVID-19 vaccine formulation, BCG:CoVac, that when delivered as a single dose induces potent SARS-CoV-2-specific immunity in mice, particularly the generation of high-titre, anti-viral neutralising antibodies. This sterilising immunity is based on plaque assays that have a limit of detection of 40 PFU that detects live virus) and is strong supported by the collective data of the complete lack of clinical signs, low weight loss and levels of inflammatory cells and cytokines/chemokines in the airways and lung tissue. Encouragingly, the level of immune responses observed (particularly the generation of neutralising antibodies) is equivalent to or exceeds immunity elicited by approved COVID-19 vaccines, when these candidate vaccines were tested in the murine model^23–25^. BCG:CoVac may have the additional advantage of inducing protection against other respiratory infections for which BCG is known to induce some level of protective immunity, including future pandemic viruses^12^. In addition, the possibility that prior BCG exposure may impart protection against severe COVID-19^26^, which is currently under evaluation through numerous randomised control studies^9^, raises the possibility that a BCG-based vaccine could afford protection against SARS-CoV-2 escape mutants or new pandemic coronavirus that may emerge. Indeed our data demonstrate that BCG:CoVac can neutralise two of the key SARS-CoV-2 VOCs, namely B.1.1.7 and B.1351. BCG:CoVac could also provide additional benefit in countries where BCG is part of childhood immunisation programmes for the control of TB, based on recent findings that repeat BCG vaccination significantly reduced rates of *M. tuberculosis* infection^27^, suggesting this vaccination regimen could provide ‘dual’ protection against both COVID-19 and TB.

An advantage of our vaccine approach is the use of alum to potentiate immune responses, particularly the generation of NAbs after vaccination. Alum is a low cost, globally accessible vaccine adjuvant with an excellent safety record in humans^28^. The relative paucity of IFN-γ-secreting T cells observed after Alum^SpK^ vaccination corresponds with that previously seen with alum-precipitated vaccines using the spike protein^29^ and is consistent with the known preferential priming of Th2-type immunity by alum-based adjuvants^30^. The ability of BCG:CoVac to induce strong Th1 immunity is due to the adjuvant effect of BCG components to induce such responses^31^. This has clear importance, as T cell responses in recovering COVID-19 patients are predominately Th1-driven^32^, expression of IFN-γ was lower in severe COVID-19 cases compared to mild ones^33^ and the development of Th2 immunity correlated with VAERD^34^. We also observed only background levels of the inflammatory cytokines IL-17 and TNF after BCG:CoVac delivery, suggesting reduced levels of potentially deleterious, circulating inflammatory cytokines. Heightened expression of IL-17 correlates with severe COVID-19 disease^35^, while neutralising IL-17 has been suggested as a possible therapy to treat acute respiratory distress syndrome in SARS-CoV-2-infected individuals^36^. In addition, the development of VAERD is also associated with Th17 immunity^37^.

Our study contributes to defining correlates of immunity in animal models that could be applied to fast-track the development of next-generation COVID-19 vaccines. A single dose of BCG:CoVac was sufficient to clear infectious SARS-CoV-2 from the lungs of K18-hACE2 mice, with no signs of clinical disease during the infection time-course, which is otherwise lethal (Fig. 4). Our findings are in agreement with previous reports that demonstrate that these mice succumb rapidly to infection, indicating that the level of NAbs elicited by BCG:CoVac is sufficient to clear SARS-CoV-2 infection in this model^20,38^. While NAb levels are known to correlate with efficacy of COVID-9 vaccines in humans^18^, other immune parameters may play an important role. Accordingly, we observed that high NAb levels in blood corelated with strong induction of GC and plasma B cells in lymph nodes, as well as heightened levels of Tfh cells. In COVID-19 convalescent individuals, the presence of memory B cells and Tfh are closely associated with NAb activity^38,39^. Further, although BCG vaccination alone did not reduce lung viral load, mice immunised with this vaccine did show an intermediate level of both weight loss and cytokine/chemokine induction during SARS-CoV-2 infection, suggesting a possible ‘dampening’ of host inflammatory responses (Fig. 4 and Supplementary Fig. 2). Such an effect was seen in a human challenge model using the live-attenuated yellow fever vaccine, where BCG reduced circulating pro-inflammatory cytokines^40^. It will be of particular interest to see the outcome of ongoing clinical trials to determine if BCG vaccination can reduce COVID-19 incidence and severity^9^.

In conclusion, we describe a COVID-19 vaccine strategy based on the existing BCG vaccine, that would be broadly applicable for all populations susceptible to SARS-CoV-2 infection. Of particular note, this strategy could be readily incorporated into current vaccine schedules in countries where BCG is currently used. Further assessment in humans will determine if BCG:CoVac can impart protective immunity against not only SARS-CoV-2, but also other respiratory infections for which BCG has known efficacy.

## Methods

### Bacterial culture

*M. bovis* BCG (strain Pasteur) was grown at 37 °C in Middlebrook 7H9 media (Becton Dickinson, BD, New Jersey, USA) supplemented with 0.5% glycerol, 0.02% Tyloxapol and 10% albumin-dextrose-catalase (ADC) or on solid Middlebrook 7H11 media (BD) supplemented with oleic acid–ADC. To prepare single cell suspensions, cultures in exponential phase (OD_600_ = 0.6) were washed in PBS, passaged 10 times through a 27 G syringe, briefly sonicated and centrifuged at low speed for 10 min to remove residual bacterial clumps. BCG suspensions were frozen at −80 °C in PBS supplemented with 20% glycerol, and colony forming units (CFU) for vaccination enumerated on supplemented Middlebrook 7H11 agar plates.

### Ethics statement

All mouse experiments were performed according to ethical guidelines as set out by the Sydney Local Health District (SLHD) Animal Ethics and Welfare Committee, which adhere to the Australian Code for the Care and Use of Animals for Scientific Purposes (2013) as set out by the National Health and Medical Research Council of Australia. SARS-CoV-2 mouse infection experiments were approved by the SLHD Institutional Biosafety Committee. COVID-19 patients were recruited through Royal Prince Alfred Hospital (RPA) Virtual, a virtual care system enabling remote monitoring of patients. The study protocol was approved by the RPA ethics committee (Human ethics number X20-0117 and 2020/ETH00770) and by the participants’ written consent. COVID-19 Patients (age range 24–60 years old) had samples taken 7–49 days after the first positive swab and were predominately asymptomatic (73%) or had mild disease at the time of sampling (27%). All associated procedures were performed in accordance with approved guidelines.

### Immunisation

Female C57BL/6 (6–8 weeks of age) purchased from Australian BioResources (Moss Vale, Australia) or hemizygous male K18-hACE2 mice bred in-house^41^ were housed at the Centenary Institute in specific pathogen-free conditions. SARS-CoV-2 full-length spike stabilised, trimeric protein (SpK) was expressed in EXPI293F™ cells and purified as described previously^42^. Mice (*n* = 3–4) were vaccinated subcutaneously (100 μl total) in the footpad (s.c) with 5 × 10^5^ CFU of BCG alone, 5 μg of SpK combined with either BCG (BCG^SpK^) or 100 μg of Alhydrogel (Alum) (Invivogen, California, USA, Alum^SpK^), or a combination of BCG (5 × 10^5^ CFU), SpK (5 μg) and Alyhydrogel (100 μg) (BCG:CoVac). Some mice were boosted three weeks after the first vaccination with 5 μg of SpK combined with 100 μg of Alhyhdrogel. Mice were bled every 2 weeks after the first immunisation (collected in 10 μl of Heparin 50,000 U/ml). Plasma was collected after centrifugation at 300×*g* for 10 min and remaining blood was resuspended in 1 mL of PBS Heparin 20 U/mL, stratified on top of Histopaque 10831 (Sigma-Aldrich, Missouri, USA) and the PBMC layer collected after gradient centrifugation.

### Flow cytometry assays

Popliteal lymph nodes were collected at day 7 post immunisation, and single cell suspensions were prepared by passing them through a 70 μm sieve. To assess specific B cell responses, 2 × 10^6^ cells were surface stained with Fixable Blue Dead Cell Stain (Life Technologies) and Spike-AF647 (1 μg) and antibodies are described in Table S1. To assess T cell responses, 2 × 10^6^ lymph node cells were stained with the antibodies as in Table S1 and cells fixed and permeabilized using the eBioscience fixation/permeabilization kit (ThermoFischer) according to the manufacturer’s protocol and intracellular staining was performed using anti-BCL-6-AF647 (clone K112-91, 1:100, BD, cat#561525).

To assess SpK-specific cytokine induction by T cells, murine PBMCs were stimulated for 4 h with SpK (5 μg/mL) and then supplemented with Protein Transport Inhibitor cocktail (Life Technologies, California, USA) for a further 10–12 h. Cells were surface stained with Fixable Blue Dead Cell Stain (Life Technologies) and the marker-specific fluorochrome-labelled antibodies as indicated in Table S1. Cells were then fixed and permeabilized using the BD Cytofix/Cytoperm^TM^ kit according to the manufacturer’s protocol. Intracellular staining was performed using the antibodies in Table S1. All samples were acquired on a BD LSR-Fortessa (BD) or a BD-LSRII and assessed using FlowJo^TM^ analysis software v10.6 (Treestar, USA). Absolute cell numbers of each sample was determined by flow cytometry using Trucount beads (BD #Cat 340334). Total cell numbers for each cell subset were calculated by multiplying the absolute cell number with the percentage of that particular subset relative to the total lymphocyte gate. Gating strategies are shown in Supplementary Fig. 3 (T cells), Supplementary Fig. 4 (B cells) and Supplementary Fig. 5 (T follicular helper cells).

### Antibody ELISA

Microtitration plates (Corning, New York, USA) were incubated overnight with 1 µg/mL SpK at room temperature (RT), blocked with 3% BSA and serially diluted plasma samples were added for 1 h at 37 °C. Plates were washed and biotinylated polyclonal goat anti-mouse IgG1 (1:50,000, abcam Cambridge, UK, cat#ab97238), polyclonal goat anti-mouse IgG2c (1:10,000, Abcam, cat# ab97253), or polyclonal goat anti-mouse IgG (1:350,000, clone abcam cat#ab6788) added for 1 h at RT. After incubation with streptavidin-HRP (1:30,000, abcam, cat#405210) for 30 min at RT, binding was visualised by addition of tetramethyl benzene (Sigma-Aldrich). The reaction was stopped with the addition of 2 N H_2_SO_4_ and absorbances were measured at 450 nm using a M1000 pro plate reader (Tecan, Männedorf, Switzerland). End point titres were calculated as the dilution of the sample that reached the average of the control serum ±3 standard deviations.

### High-content live SARS-CoV-2 neutralization assay

High-content fluorescence microscopy was used to assess the ability of sera/plasma to inhibit SARS-CoV-2 infection and the resulting cytopathic effect in live permissive cells (VeroE6). Sera were serially diluted and mixed in duplicate with an equal volume of 1.5 × 10^3^ TCID_50_/mL virus solution (B.1.319) or 1.25 × 10^4^ TCID_50_/mL virus solution (A2.2, B.1.1.7, B.1.351). After 1 h of virus-serum coincubation at 37 °C, 40 μL were added to equal volume of freshly-trypsinised VeroE6 cells in 384-well plates (5 × 10^3^/well). After 72 h, cells were stained with NucBlue (Invitrogen, USA) and the entire well surface was imaged with InCell Analyzer 2500 (Cytiva). Nuclei counts were obtained for each well with InCarta software (Cytiva), as proxy for cell death and cytopathic effect resulting from viral infection. Counts were compared between convalescent sera, mock controls (defined as 100% neutralisation), and infected controls (defined as 0% neutralisation) using the formula; % viral neutralisation = (*D*−(1−*Q*)) × 100/*D*, where *Q* = nuclei count of sample normalised to mock controls, and *D* = 1−*Q* for average of infection controls. The cut-off for determining the neutralisation endpoint titre of diluted serum samples was set to ≥50% neutralisation.

### SARS-CoV-2 challenge experiments

Male hemizygous K18-hACE2 mice were transported to the PC3 facility in the Centenary Institute for SARS-CoV-2 infection. Mice were anaesthetised with isoflurane followed by intranasal challenge with 10^3^ PFU SARS-CoV-2 (VIC01/2020) in a 30 µL volume. Following infection, mice were housed in the IsoCage N biocontainment system (Tecniplast, Italy) and were given access to standard rodent chow and water *ad libitum*. Mice were weighed and monitored daily, with increased frequency of monitoring when mice developed symptoms. At day 6 post-infection, mice were euthanised with intraperitoneal overdose of pentobarbitone (Virbac, Australia). Blood was collected via heart bleed, allowed to coagulate at RT and centrifuged (10,000×*g*, 10 min) to collect serum. Multi-lobe lungs were tied off and BALF was collected from the single lobe via lung lavage with 1 mL HANKS solution using a blunted 19-gauge needle inserted into the trachea. BALF was centrifuged (300 *g*, 4 °C, 7 min), and supernatants collected and snap frozen. Cell pellets were treated with 200 µL Red Blood Cell Lysis Buffer (ThermoFisher, USA) for 5 min, followed by addition of 700 µL HANKS solution to inactivate the reaction and then centrifuged again. Cell pellets were resuspended in 160 µL HANKS solution and enumerated using a haemocytometer (Sigma-Aldrich, USA). Multi-lobe lungs were collected and cut into equal thirds, before snap freezing on dry ice. Lung homogenates were prepared fresh, with multi-lobe lungs placed into a gentleMACS C-tube (Miltenyi Biotec, Australia) containing 2 mL HANKS solution. Tissue was homogenised using a gentleMACS tissue homogeniser, after which homogenates were centrifuged (300×*g*, 7 min) to pellet cells, followed by collection of supernatants for plaque assays and cytokine/chemokine measurements. The single lobe lung was perfused with 0.9% NaCl solution via the heart, followed by inflation with 0.5 mL 10% neutral buffered formalin through the trachea, and placed into a tube containing 10% neutral buffered formalin. Following fixation for at least 2 weeks, single lobes were transported to a PC2 facility where they were paraffin-embedded, sections cut to 3 µm thickness using a Leica microtome (Leica, Germany) and then stained using Quick Dip Stain Kit (Modified Giemsa Stain) protocol as per manufacturer’s instructions (POCD Scientific, Australia). Inflammatory cells in single lobe lungs were counted using a Zeiss Axio Imager.Z2 microscope with a ×40 objective (Zeiss, Germany).

### Plaque assays

VeroE6 cells (CellBank Australia, Australia) were grown in Dulbecco’s Modified Eagles Medium (Gibco, USA) supplemented with 10% heat-inactivated foetal bovine serum (Sigma-Aldrich, USA) at 37 °C/5% CO_2_. For plaque assays, cells were placed into a 24-well plate at 1.5 × 10^5^ cells/well and allowed to adhere overnight. The following day, virus-containing samples were serially diluted in Modified Eagles Medium (MEM), cell culture supernatants removed from the VeroE6 cells and 250 µL of virus-containing samples was added to cell monolayers. Plates were incubated and gently rocked every 15 min to facilitate viral adhesion. After 1 h, 250 µL of 0.6% agar/MEM solution was gently overlaid onto samples and placed back into the incubator. At 72 h post-infection, each well was fixed with an equal volume of 8% paraformaldehyde solution (4% final solution) for 30 min at RT, followed by several washes with PBS and incubation with 0.025% crystal violet solution for 5 min at RT to reveal viral plaques.

### Cytometric bead arrays

Cytometric bead arrays (CBAs) were performed as per the manufacturer’s instructions (Becton Dickinson, USA). Briefly, a standard curve for each analyte was generated using a known standard supplied with each CBA Flex kit. For each sample, 10 µL was added to a well in a 96-well plate, followed by incubation with 1 µL of capture bead for each analyte (1 h, RT, in the dark). Following capture, 1 µL of detection bead for each analyte was added to each well, followed by incubation (2 h, RT, in the dark). Samples were then fixed overnight in an equal volume of 8% paraformaldehyde solution (4% final solution). The following day, samples were transferred to a new 96-well plate and then transported to the PC2 facility for a second round of fixation. Samples were examined using a BD LSR Fortessa equipped with a High-Throughput Sampler (HTS) plate reader.

### Mycobacterium tuberculosis aerosol challenge

Eight weeks after the last vaccination mice were infected with *M. tuberculosis* H37Rv via the aerosol route using a Middlebrook airborne infection apparatus (Glas-Col, IN, USA) with an infective dose of ~100 viable bacilli. Four weeks later, the lungs and spleen were harvested, homogenised, and plated after serial dilution on supplemented Middlebrook 7H11 agar plates. Colonies forming units (CFU) were determined 3 weeks later and expressed as log_10_ CFU.

### Statistical analysis

The significance of differences between experimental groups was evaluated by one-way analysis of variance (ANOVA), with pairwise comparison of multi-grouped data sets achieved using Tukey’s or Dunnett’s post-hoc test. Where required, log transformation was used to obtain normal distribution and variance homogeneity prior to analysing data. Differences were considered statistically significant when *p* ≤ 0.05.

### Reporting summary

Further information on research design is available in the Nature Research Reporting Summary linked to this article.

## Supplementary information

Supplementary Information

Reporting Summary

## Data Availability

The datasets generated during and/or analysed during the current study are available from the corresponding author on reasonable request.
