# Supplementary material for: A single dose, BCG-adjuvanted COVID-19 vaccine provides sterilising immunity against SARS-CoV-2 infection
Source: NPJ Vaccines. 2021 Nov 30;6:143. doi: 10.1038/s41541-021-00406-4 (PMC8633321; doi:10.1038/s41541-021-00406-4)
Supplement: Supplementary file 1 — Supplementary Information [file 41541_2021_406_MOESM1_ESM.pdf]

A single dose, BCG-adjuvanted COVID-19 vaccine provides sterilizing immunity against SARS-CoV-2 infection

SUPPLEMENTARY INFORMATION

Claudio Counoupas[1,2], Matt D. Johansen[3], Alberto O. Stella[4], Duc H. Nguyen[3], Angela L. Ferguson[1,2] Anupriya Aggarwal[4], Nayan D. Bhattacharyya[2], Alice Grey[5], Owen Hutchings[6], Karishma Patel[7], Rezwan Siddiquee[7], Erica L. Stewart[1], Carl G. Feng[2], Nicole G. Hansbro[3], Umaimainthan Palendira[1], Megan C. Steain[2], Bernadette M. Saunders[3], Jason K. K. Low[7], Joel P. Mackay[7], Anthony D. Kelleher[4], Warwick J. Britton[2,5], Stuart G Turville[4], Philip M. Hansbro[3*], James A. Triccas[1,8*]

[1]School of Medical Sciences, Faculty of Medicine and Health, The University of Sydney, Camperdown, NSW, Australia.

[2]Tuberculosis Research Program at the Centenary Institute, The University of Sydney, Sydney, NSW, Australia.

[3]Centre for Inflammation, Centenary Institute and University of Technology Sydney, Faculty of Science, School of Life Sciences, Sydney, NSW, Australia.

[4]Kirby Institute, University of New South Wales, Sydney, NSW, Australia.

[5]Department of Clinical Immunology, Royal Prince Alfred Hospital, Sydney, NSW, Australia.

[6]RPA Virtual Hospital, Sydney Local Health District, Sydney, NSW, Australia.

[7]School of Life and Environmental Sciences, The University of Sydney, Sydney, NSW 2006.

[8]Charles Perkins Centre and Marie Bashir Institute for Infectious Diseases and Biosecurity, The University of Sydney, Camperdown, NSW, Australia.

*Correspondence to James A. Triccas (jamie.triccas@sydney.edu.au); Philip Hansbro (Philip.Hansbro@uts.edu.au)

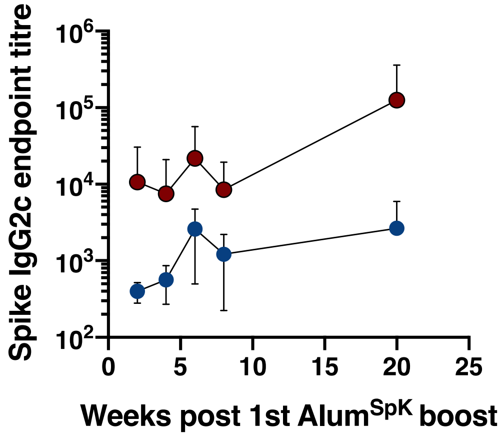

Supplementary Figure 1. BCG promotes spike-specific antibody reponses. Mice were vaccinated s.c with BCG ($5x10^5$ CFU) and 12 week later were vaccinated twice, 3 weeks apart with s.c with SARS-CoV-2 spike protein (5 μg) formulated in alum (100 μg; Alum^{SpK}). At the indicated timepoints the titre of spike-specific IgG2c in sera was determined by ELISA.

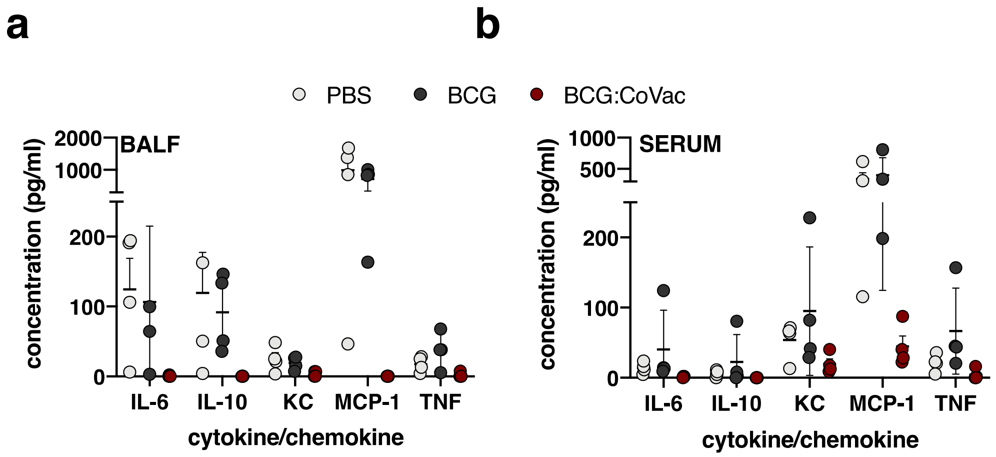

Supplementary Figure 2. Single dose BCG:CoVac prevents the development of clinical disease in SARS-CoV-2 infected K18-hACE2 mice. a, Mice were immunised with sham (PBS), BCG or BCG:CoVac 21 days prior to challenge with 10^3 PFU SARS-CoV-2. Cytokine/chemokine levels were determined in BALF (**a**) and serum (**b**) by cytometric bead array 6 days after challenge.

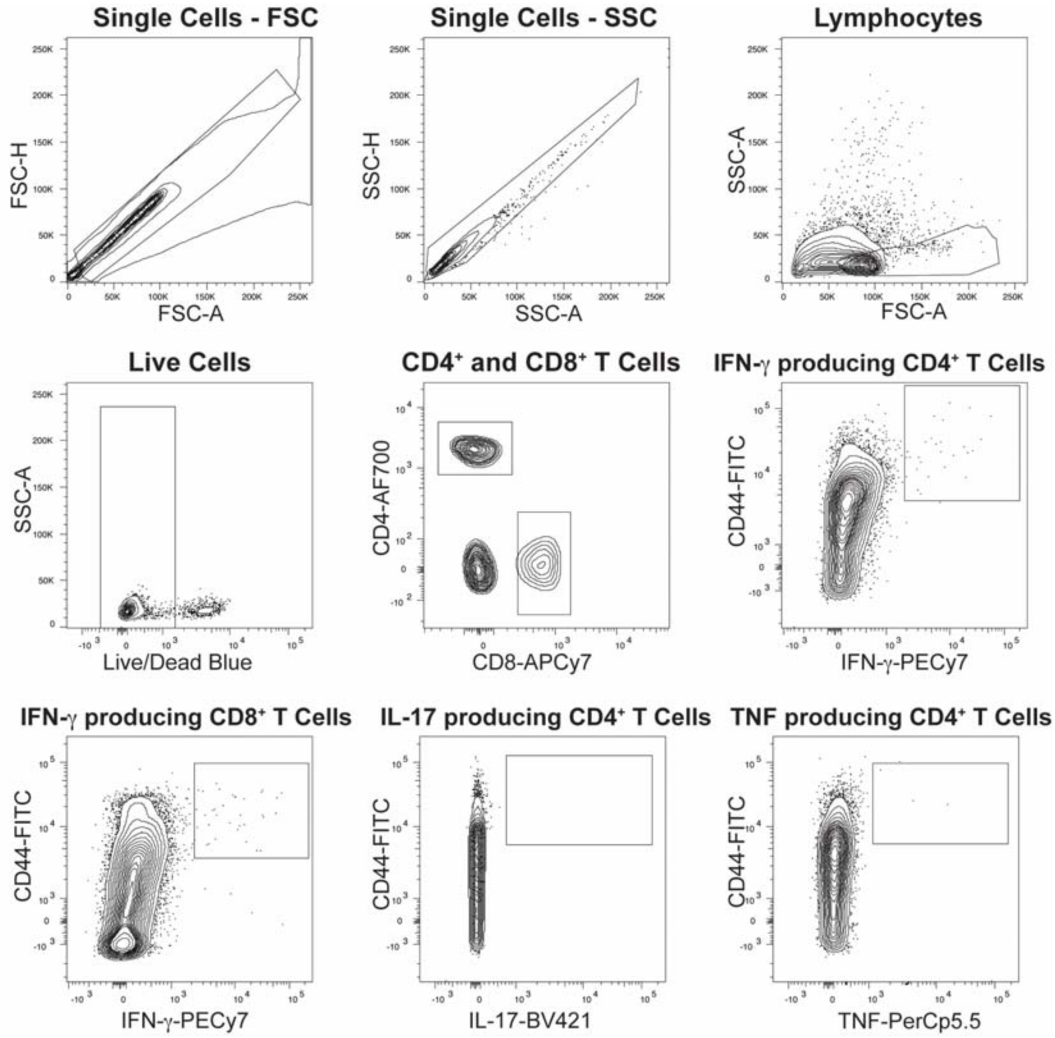

Supplementary Figure 3. Gating strategy for cytokine producing CD4$^+$ and CD8$^+$ T cells from PBMCs. Single events were selected using FSC-H/FSC-A and SSC-A/SSC-H. Lymphocytes populations were gated by SSC-A and FSC-A plots. Dead cells were excluded and viable CD4$^+$ and CD8$^+$ T cells were gated for further analysis of expression of different cytokines (IFN-γ, IL-17, TNF).

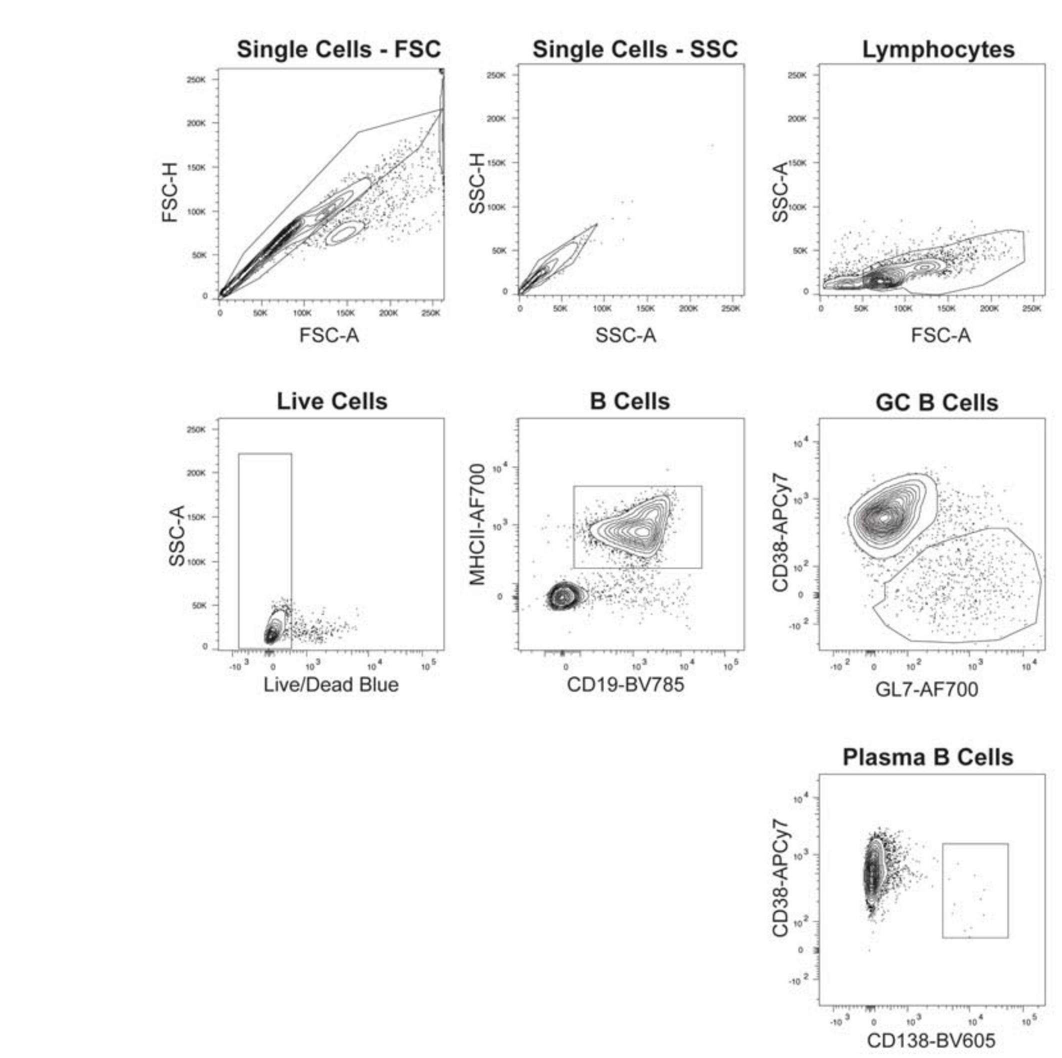

Supplementary Figure 4. Gating strategy for Germinal Center B cells and Plasma B Cells.

Single events were selected using FSC-H/FSC-A and SSC-A/SSC-H. Lymphocytes populations were gated by SSC-A and FSC-A plots. Dead cells were excluded and viable B cells were gated on the double expression of MHCII and CD19. GC B cells were gated based on their expression of GL7 and lacking expression of CD38. Plasma B cells were gated based on their expression of CD138.

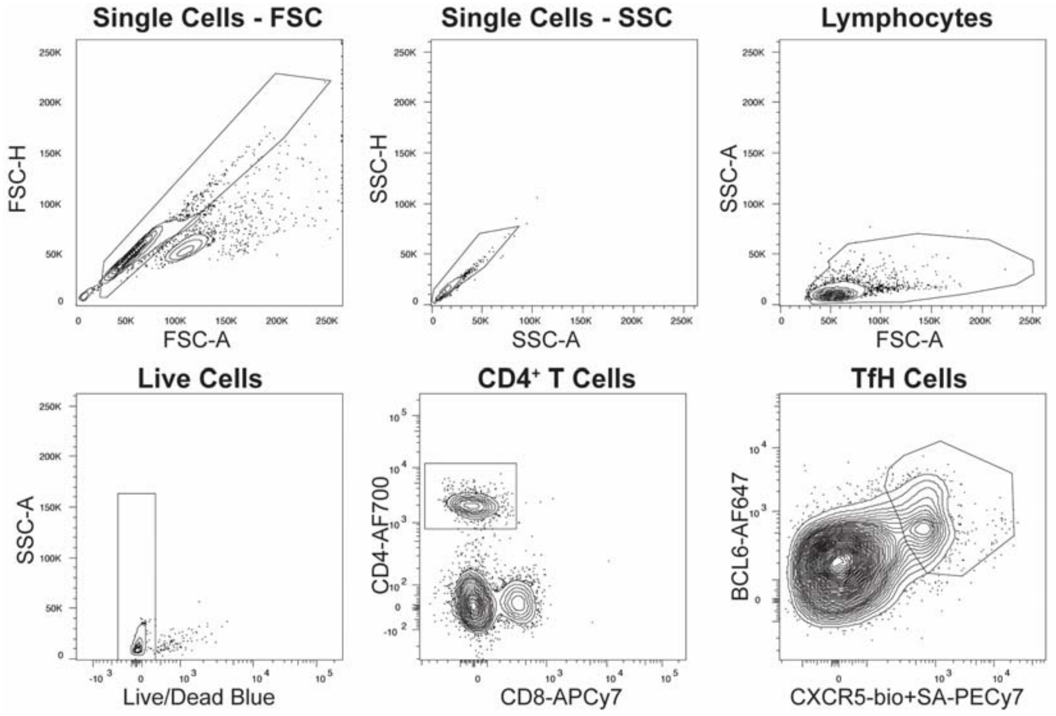

Supplementary Figure 5. Gating strategy for T follicular helper cells in the lymph node. Single events were selected using FSC-H/FSC-A and SSC-A/SSC-H. Lymphocytes populations were gated by SSC-A and FSC-A plots. Dead cells were excluded and viable $CD4^+$ T cells were gated for further analysis of expression BCL6 and CXCR5, double positive cells for this markers were considered Tfh cells.

Supplementary Table 1: Flow cytometry antibodies used in this study

T cell panel

Marker	Fluorophore	Location	Clone	Dilution	Company	Cat. #
Live/Dead	Blue	Surface		1:300	ThermoFisher	L23105
Fc Block	purified	Surface	2462	1:300	BD	553141
CD4	AF700	Surface	RM414	1:200	BD	557956
CD44	BV605	Surface	IM7	1:300	BD	563058
CD8	APC-Cy7	Surface	53-6.7	1:200	BD	557654
CXCR5	Biotin	Surface	2G8	1:100	BD	551960
PD-1	BV711	Surface	29F.1A12	1:200	Biolegend	135231
Streptavidin	PE-Cy7	Surface		1:200	BD	557598
BCL-6	AF647	Intracellular	K112-91	1:200	BD	561525
CD19	BV785	Surface	1D3	1:200	BD	563333
IFN-γ	PECy7	Intracellular	XMG1-2	1:200	BD	557649
IL-17	PB	Intracellular	TC11-	1:200	BioLegend	506918
IL-2	PE	Intracellular	JES6-5H4	1:200	BD	554428
TNF	PerCP-Cy5.5	Intracellular	MP6-	1:200	BD	560659

B cell panel

Marker	Fluorophore	Location	Clone	Dilution	Company	Cat. #
Live/Dead	Blue	Surface		1:300	ThermoFisher	L23105
Fc Block	purified	Surface	2462	1:300	BD	553141
CD19	BV785	Surface	1D3	1:200	BD	563333
Spike	AF647	Surface		1:50		
GL7	AF488	Surface	GL7	1:200	Biolegend	144612
MHCII	AF700	Surface	M5/114.15.2	1:200	Biolegend	107622
CD138	BV605	Surface	281-2	1:200	Biolegend	142516
CD38	APC-Cy7	Surface	90	1:200	Biolegend	102728
IgM	BV421	Surface	RMM-1	1:200	Biolegend	406518
IgD	PerCP5.5	Surface	11-26c.2a	1:200	BD	564273
