# Supplementary material for: A single dose, BCG-adjuvanted COVID-19 vaccine provides sterilising immunity against SARS-CoV-2 infection
Source: NPJ Vaccines. 2021 Nov 30;6:143. doi: 10.1038/s41541-021-00406-4 (PMC8633321; doi:10.1038/s41541-021-00406-4)
Supplement: Supplementary file 2 — Reporting Summary [file 41541_2021_406_MOESM2_ESM.pdf]

nature portfolio

Corresponding author(s): James Triccas and Phillip Hansbro

Last updated by author(s): Aug 30, 2021

Reporting Summary

Nature Portfolio wishes to improve the reproducibility of the work that we publish. This form provides structure for consistency and transparency in reporting. For further information on Nature Portfolio policies, see our Editorial Policies and the Editorial Policy Checklist.

Statistics

For all statistical analyses, confirm that the following items are present in the figure legend, table legend, main text, or Methods section.

n/a	Confirmed	
☐	☒	The exact sample size (*n*) for each experimental group/condition, given as a discrete number and unit of measurement
☐	☒	A statement on whether measurements were taken from distinct samples or whether the same sample was measured repeatedly
☐	☒	The statistical test(s) used AND whether they are one- or two-sided<br>*Only common tests should be described solely by name; describe more complex techniques in the Methods section.*
☒	☐	A description of all covariates tested
☐	☒	A description of any assumptions or corrections, such as tests of normality and adjustment for multiple comparisons
☐	☒	A full description of the statistical parameters including central tendency (e.g. means) or other basic estimates (e.g. regression coefficient) AND variation (e.g. standard deviation) or associated estimates of uncertainty (e.g. confidence intervals)
☐	☒	For null hypothesis testing, the test statistic (e.g. *F*, *t*, *r*) with confidence intervals, effect sizes, degrees of freedom and *P* value noted<br>*Give P values as exact values whenever suitable.*
☒	☐	For Bayesian analysis, information on the choice of priors and Markov chain Monte Carlo settings
☒	☐	For hierarchical and complex designs, identification of the appropriate level for tests and full reporting of outcomes
☒	☐	Estimates of effect sizes (e.g. Cohen's *d*, Pearson's *r*), indicating how they were calculated

Our web collection on statistics for biologists contains articles on many of the points above.

Software and code

Policy information about availability of computer code

Data collection	FACS DIVA<br>Tecan iControl software<br>InCarta software
Data analysis	GraphPad Prism v.7<br>FlowJo v10.6

For manuscripts utilizing custom algorithms or software that are central to the research but not yet described in published literature, software must be made available to editors and reviewers. We strongly encourage code deposition in a community repository (e.g. GitHub). See the Nature Portfolio guidelines for submitting code & software for further information.

Data

Policy information about availability of data

All manuscripts must include a data availability statement. This statement should provide the following information, where applicable:

- Accession codes, unique identifiers, or web links for publicly available datasets
- A description of any restrictions on data availability
- For clinical datasets or third party data, please ensure that the statement adheres to our policy

The datasets generated during and/or analysed during the current study are available from the corresponding author on reasonable request.

Field-specific reporting

Please select the one below that is the best fit for your research. If you are not sure, read the appropriate sections before making your selection.

☒ Life sciences ☐ Behavioural & social sciences ☐ Ecological, evolutionary & environmental sciences

For a reference copy of the document with all sections, see nature.com/documents/nr-reporting-summary-flat.pdf

Life sciences study design

All studies must disclose on these points even when the disclosure is negative.

Sample size	For all experiments, we have determined the number of animals required based on the ability to detect a 40% difference between groups, a significance of $p<0.05$ and 80% power to reject the null hypothesis given the alternative hypothesis is true.
Data exclusions	No Data was excluded
Replication	All experiments were performed at least twice, except where indicated
Randomization	Allocation of mice to experimental groups was randomized upon their arrival to the animal facility.
Blinding	Investigators were not blinded to animal groups due to the nature of vaccine delivery. Groups of mice were allocated to receive particular methods of vaccine delivery and vaccine doses which remained consistent throughout the experiment.

Reporting for specific materials, systems and methods

We require information from authors about some types of materials, experimental systems and methods used in many studies. Here, indicate whether each material, system or method listed is relevant to your study. If you are not sure if a list item applies to your research, read the appropriate section before selecting a response.

Materials & experimental systems

n/a	Involved in the study	
☐	☒	Antibodies
☐	☒	Eukaryotic cell lines
☒	☐	Palaeontology and archaeology
☐	☒	Animals and other organisms
☒	☐	Human research participants
☒	☐	Clinical data
☒	☐	Dual use research of concern

Methods

n/a	Involved in the study	
☒	☐	ChIP-seq
☐	☒	Flow cytometry
☒	☐	MRI-based neuroimaging

Antibodies

Antibodies used

Live/Dead Blue Surface 1:300 ThermoFisher L23105
Fc Block purified Surface 2462 1:300 BD 553141
CD4 AF700 Surface RM414 1:200 BD 557956
CD44 BV605 Surface IM7 1:300 BD 563058
CD8 APC-Cy7 Surface 53-6.7 1:200 BD 557654
CXCR5 Biotin Surface 2G8 1:100 BD 551960
PD-1 BV711 Surface 29F.1A12 1:200 Biolegend 135231
Streptavidin PE-Cy7 Surface 1:200 BD 557598
BCL-6 AF647 Intracellular K112-91 1:200 BD 561525
CD19 BV785 Surface 1D3 1:200 BD 563333
IFN-gamma PECy7 Intracellular XMG1-2 1:200 BD 557649
IL-17 PB Intracellular TC11-18H10.1 1:200 BioLegend 506918
IL-2 PE Intracellular JES6-5H4 1:200 BD 554428
TNF PerCP-Cy5.5 Intracellular MP6-XT22 1:200 BD 560659
CD19 BV785 Surface 1D3 1:200 BD 563333
Spike AF647 Surface 1:50
GL7 AF488 Surface GL7 1:200 Biolegend 144612
MHCII AF700 Surface M5/114.15.2 1:200 Biolegend 107622
CD138 BV605 Surface 281-2 1:200 Biolegend 142516
CD38 APC-Cy7 Surface 90 1:200 Biolegend 102728
IgM BV421 Surface RMM-1 1:200 Biolegend 406518
IgD PerCP5.5 Surface 11-26c.2a 1:200 BD 564273

Validation	Antibodies were validated before use based on the conditions suggested by the manufacturer.

Eukaryotic cell lines

Policy information about cell lines

Cell line source(s)	ATCC
Authentication	cell lines were not authenticated
Mycoplasma contamination	all cell lines were negative for mycoplasma
Commonly misidentified lines (See ICLAC register)	N/A

Animals and other organisms

Policy information about studies involving animals; ARRIVE guidelines recommended for reporting animal research

Laboratory animals	Female C57BL/6 (6-8 weeks of age) or male K18-hACE2 mice (6-8 weeks of age)
Wild animals	Study did not involve wild animals
Field-collected samples	Study did not involve samples collected on the field
Ethics oversight	Sydney Local Health District (SLHD) Animal Ethics and Welfare Committee approved the ethic protocol of the study.

Note that full information on the approval of the study protocol must also be provided in the manuscript.

Flow Cytometry

Plots

Confirm that:

☒ The axis labels state the marker and fluorochrome used (e.g. CD4-FITC).

☒ The axis scales are clearly visible. Include numbers along axes only for bottom left plot of group (a 'group' is an analysis of identical markers).

☒ All plots are contour plots with outliers or pseudocolor plots.

☒ A numerical value for number of cells or percentage (with statistics) is provided.

Methodology

Sample preparation	Popliteal lymph nodes were collected at day 7 post immunization, and single cell suspensions were prepared by passing them through a 70 µm sieve. To assess specific B cell responses, 2x106 cells were surface stained with Fixable Blue Dead Cell Stain (Life Technologies) and Spike-AF647 (1 µg) and antibodies are described in Table S1. To assess T cell responses, 2x106 lymph node cells were stained with the antibodies as in Table S1 and cells fixed and permeabilized using the eBioscience fixation/permeabilization kit (ThermoFischer) according to the manufacturer's protocol and intracellular staining was performed using anti-BCL-6-AF647 (clone K112-91, 1:100, BD, cat#561525). To assess SpK-specific cytokine induction by T cells, murine PBMCs were stimulated for 4 hrs with SpK (5 µg/mL) and then supplemented with Protein Transport Inhibitor cocktail (Life Technologies, California, USA) for a further 10-12 hrs. Cells were surface stained with Fixable Blue Dead Cell Stain (Life Technologies) and the marker-specific fluorochrome-labeled antibodies as indicated in Table S1. Cells were then fixed and permeabilized using the BD Cytofix/CytopermTM kit according to the manufacturer's protocol. Intracellular staining was performed using the antibodies in Table S1. All samples were acquired on a BD LSR-Fortessa (BD) or a BD-LSRII and assessed using FlowJoTM analysis software v10.6 (Treestar, USA). Absolute cell numbers of each sample was determined by flow cytometry using Trucount beads (BD #Cat 340334). Total cell numbers for each cell subset were calculated by multiplying the absolute cell number with the percentage of that particular subset relative to the total lymphocyte gate.
Instrument	BD-LSRII or BD LSR Fortessa
Software	FACS DIVA
Cell population abundance	At least one million events were acquired to account for rare populations.
Gating strategy	Gating strategy available in the Supplementary Material

☒ Tick this box to confirm that a figure exemplifying the gating strategy is provided in the Supplementary Information.
